# Complete chloroplast genome sequence of *Stauntonia hexaphylla* (Ranunculales: Lardizabalaceae), a species endemic to Korea

**DOI:** 10.1080/23802359.2021.1885320

**Published:** 2021-03-15

**Authors:** Yonguk Kim, Jawon Shin, Dong-Wook Kim, Hak-Sung Lee, Chulyung Choi

**Affiliations:** a Jeonnam Institute of Natural Resources Research, Jeonnam, Republic of Korea; b Coscience Co. Ltd, Muan-gun, Republic of Korea

**Keywords:** Chloroplast genome, Lardizabalaceae, *Stauntonia hexaphylla*

## Abstract

The complete chloroplast (cp) genome of *Stauntonia hexaphylla*, a monotypic genus native to Korea, was determined. The whole cp genome is 158,390 bp in size, containing a large single-copy (LSC) region of 87,115 bp and a small single-copy region (SSC) of 18,928 bp, separated by a pair of inverted repeats (IRs) of 26,174 bp. The cp genome encodes 117 genes, including 79 protein-coding, 38 tRNA-coding, and 8 rRNA-coding genes. The overall GC content is 37.8%. A phylogenetic analysis demonstrated a close relationship between *S. hexaphylla* and *S. obovatifoliola* subsp. *urophylla*. The cp genome will provide new insight into the evolution of Lardizabalaceae.

*Stauntonia hexaphylla* (Thunb.) Decne. is an evergreen climber native to Korea distributed in the coastal regions of Goheung and Haenam and intensively cultured in the Jangheung, Jeonnam province. It has been used as a traditional herbal medicine for analgesic, sedative, and diuretic properties (Kim et al. 2018). To gain insight into its evolution and facilitate genetic research into *Stauntonia*, we characterized the complete chloroplast (cp) genome of *S. hexaphylla* based on Illumina sequencing data.

Fresh leaf samples were collected from Anyang-myeon (34°39′36.0ʺN, 126°56′24.0ʺE), Jangheung County, Jeonnam Province, Republic of Korea, and deposited at the herbarium of the Jeollanamdo Institute of Natural Resources (JINR), Korea (voucher specimen number JINR00000120). Total genomic DNA was extracted from leaf samples using a modified CTAB method (Allen et al. 2006). The genome library (paired-end, PE =150 bp) was constructed at Coscience Co. Ltd. (Mokpo, South Korea) using the Illumina Nova Seq 6000 platform (Illumina Inc., San Diego, CA, USA). FastQC (v0.11.8) was used to conduct quality assessment (Andrews 2010) and all clean data was used for *de novo* assembly using SOAPdenovo2 (Luo et al. 2012) with the cp genome of the closely related species *S. obovatifoliola* subsp. *urophylla* (NC_047215) as the reference. Finally, the assembled cp genome was annotated and adjusted manually using the software Geneious v11.0.4. (Kearse et al. 2012). The complete cp genome of *S. hexaphylla* was submitted to GenBank under accession number LC595632.

The size of cp genome of *S. hexaphylla* is 158,390 bp in length including a pair of identical IRs (26,174 bp) separated by LSC (87,115 bp) and SSC (18,927 bp) regions. The overall GC content was 37.8%, whereas those of the LSC, SSC, and each IR are 37.1%, 40.6%, and 33.8%, respectively. It encodes a total of 117 genes, including 79 protein-coding genes, 38 tRNA-coding genes, and 8 rRNA-coding genes.

To confirm the phylogenetic location of *S. hexaphylla* within the family of Lardizabalaceae, a total of 10 complete cp genomes of Lardizabalaceae were obtained from Genbank, and aligned using ClustalW from Mega 7.0 (Kumar et al. 2016). *Decaisnea insignis* and *Sinofranchetia chinensis* were designated as outgroups. The phylogenetic analysis was performed using the maximum likelihood (ML) method in Mega 7.0 with the Kimura 2-parameter model and 1000 bootstrap replicates.

Phylogenetic analysis based on the complete cp genomes showed that *S. hexaphylla* was most closely related to *S. obovatifoliola* subsp. *urophylla* (Figure 1). The result shows the position of *S. hexaphylla* from Lardizabalaceae, which is consistent with previous DNA-based phylogenetic studies (Hoot et al. 1995; Wang et al. 2009). The *S. hexaphylla* genome reported in this study may provide useful genetic information for further assessing the genetic diversity in this species for conservation purpose, and may be used to better resolve the phylogenetic relationships among members of the genus *Stauntonia*.

**Figure 1.**
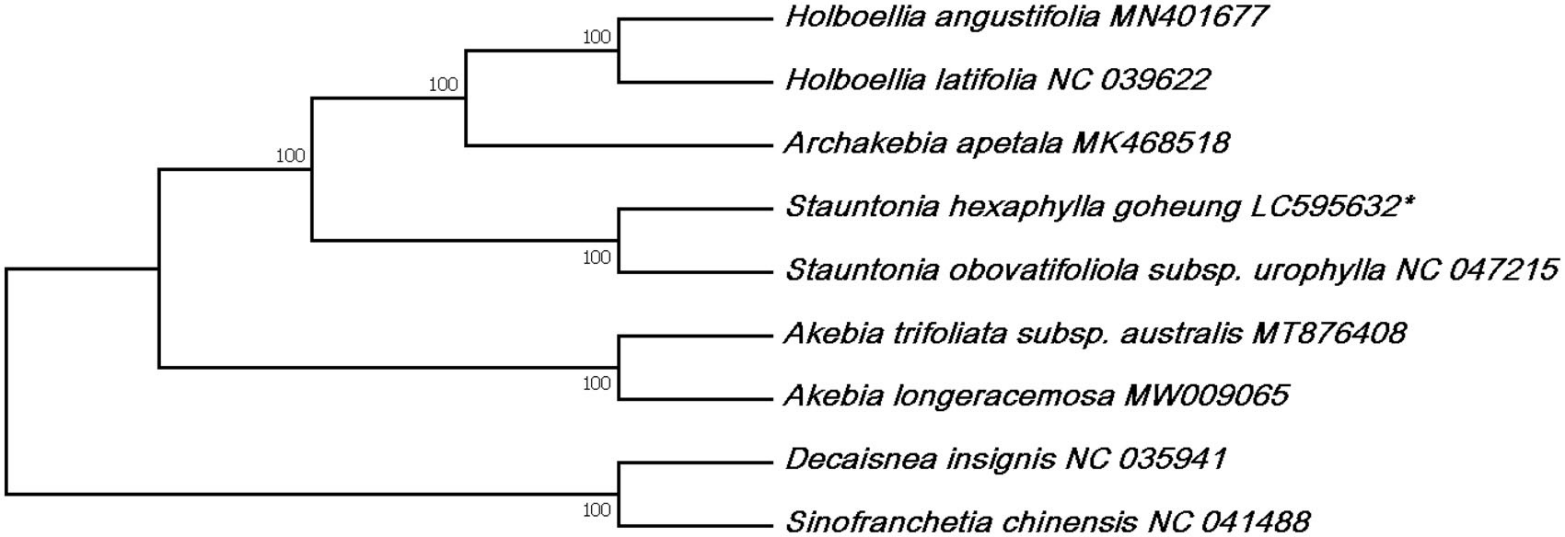
The maximum likelihood (ML) tree based on complete cp genomes of *Stauntonia hexaphylla* and 9 related Lardizabalaceae. The bootstrap values based on 1000 replicates are shown on each node. Accession numbers: *Holboellia angustifolia* MN401677*, Holboellia latifolia* NC039622*, Archakebia apetala* MK468518*, Stauntonia hexaphylla* LC595632*, Stauntonia obovatifoliola* subsp. *urophylla* NC047215, *Akebia longeracemosa* MW009065*, Akebia trifoliata* subsp. *australis* MT876408*, Akebia trifoliata* NC029427*, Decaismea insignis* NC035941, and *Sinofranchetia chinensis* NC041488.

## Data Availability

Chloroplast data supporting this study are openly available in Genbank at nucleotide database, http://www.ncbi.nlm.gov/nuccore/LC595632, Associated BioProject, https://www.ncbi.nlm.nih.gov/bioproject/PRJDB11053, BioSample accession number at https://www.ncbi.nlm.nih.gov/biosample/SAMD00271666 and Sequence Read Archive at http://trace.ddbj.nig.ac.jp/DRASearch/submission?acc=DRA011448.
